# Investigation of food safety perceptions, practices, and workplace policies among employees of pet and animal feed stores that sell and do not sell raw meat-based diets

**DOI:** 10.3389/fvets.2025.1523996

**Published:** 2025-05-21

**Authors:** Jennifer Lord, Marie Cozzarelli, Sara Lyon, Sheri Pugh, Sharon R. Thompson

**Affiliations:** ^1^Department of Biomedical and Diagnostic Sciences, College of Veterinary Medicine, University of Tennessee, Knoxville, TN, United States; ^2^Department of Public Health, University of Tennessee, Knoxville, TN, United States; ^3^Gillings School of Public Health, Epidemiology, University of North Carolina Chapel Hill, Chapel Hill, NC, United States; ^4^Center for Agriculture and Food Security and Preparedness, Department of Biomedical and Diagnostic Sciences, College of Veterinary Medicine, University of Tennessee, Knoxville, TN, United States; ^5^Tennessee Integrated Food Safety Center for Excellence, Nashville and Knoxville, TN, United States

**Keywords:** zoonotic disease, food safety, raw pet food, public health, pet store, animal feed, one health

## Abstract

**Background:**

Pet and animal feed store employees face numerous occupational health hazards, including exposure to pathogens from handling contaminated animal food products. This study aimed to (1) investigate knowledge, sanitation practices, and workplace policies related to handling animal food and treats among employees of pet food and animal feed retailers in the United States (US) and (2) determine whether differences exist between employees of stores that sell raw pet food products and those that do not.

**Methods:**

A survey evaluating sanitation practices, training, and knowledge of disease risk related to animal husbandry and food handling was distributed to pet and animal feed store employees across the US by the University of Tennessee Center for Agriculture and Food Security and Preparedness (CAFSP), part of the Tennessee Integrated Food Safety Center of Excellence. Pet and feed stores that were contacted for participation were identified by searching for stores that posted their email addresses online. Student workers and CAFSP contractors helped to identify pet and feed stores in their area and distribute surveys, which were disseminated via email, mail, and hand-delivery. Chi-square tests and Wilcoxon rank sum tests were used to assess differences between employees of stores that sold raw pet food and those that did not.

**Results:**

Two hundred and six surveys were completed by employees of pet and animal feed stores in the 15 states, located in the Southeast, Midwest, Southwest, and Western US. Overall, just 25.3% (95% CI, 19.7, 31.7%) of respondents had received training on disease risk related to handling animal food. Compared to employees of stores that did not sell raw food, those who worked at raw food retailers had significantly higher perceived risk of illness (*p* = 0.0360). They tended to report more frequent surface disinfection (*p* = 0.0054), but not handwashing (*p* = 0.0542) than those who did not sell raw food. There were no significant differences in general workplace handwashing policies (*p* = 0.7800) or those specifically related to handling animal food (*p* = 0.0517). A substantial percentage of employees of both raw food retailers (41.5%) and those that did not sell raw food (67.8%) either rarely or never provided customers with food safety information.

**Conclusion:**

Findings of this study indicate a need for ongoing training and outreach regarding food safety practices and zoonotic and foodborne disease risk among animal feed store employees. Stores should implement clear workplace hygiene policies and expand employee training and customer education to improve food safety standards and minimize disease risks.

## 1 Introduction

Employees of pet and animal feed retailers face numerous occupational health hazards, including exposure to zoonotic and foodborne pathogens (1). Transmission of infectious diseases associated with pet stores and distributors has been reported since the 1970s, and handling animals, as well as animal food items or pet treats, which may be contaminated with zoonotic pathogens, can lead to illness in humans (2–7). Employees of pet and animal feed stores have the potential to serve as an information source about disease risk and food and animal handling practices for pet owners, who are also at risk of zoonotic and foodborne illness from handling pet food (3). Appropriate food handling practices and awareness of the risk of zoonotic and foodborne illness are essential for those in contact with raw pet food products, which have not undergone heat treatment to reduce pathogen loads (8, 9). These products have become increasingly popular among dog and cat owners for supplemental feeding or as a primary diet source (9, 10). A survey of 2,337 dog and cat owners in the United States (US) conducted in 2016 reported that 38% of cat owners and 46% of dog owners had fed their pet a raw diet (11). Another survey, conducted in 2018, which included 1,295 respondents from the US, found that 62.4% of dog owners and 49.1% of cat owners fed raw foods as part or all of their pets’ diet (10).

Raw meat-based diets (RMBDs) typically consist of uncooked meat, animal by-products, and bones (8, 9). Pet owners who feed these diets tend to report doing so because they believe they are healthier for their pets (9, 11–15). However, compared to conventionally processed foods, RMBDs are more likely to be contaminated with food safety pathogens such as *Salmonella* and *Listeria monocytogenes*, which have been isolated from commercial raw pet food products in numerous studies (16–21). Dogs fed raw meat have a significantly higher incidence of fecal *Salmonella* shedding than dogs that do not consume raw meat, and may serve as a source of environmental contamination (22–24). Other bacterial pathogens, including *Escherichia coli* and *Campylobacter*, have also been isolated from raw pet food products (17–20, 25, 26).

In the United States, the Food and Drug Administration (FDA) regulates pet foods under the Federal Food, Drug & Cosmetic Act and the Food Safety Modernization Act, which focuses on prevention of contamination and other food safety hazards and requires that facilities create and implement a food safety plan (27). The FDA’s Center for Veterinary Medicine provides guidance on the manufacture and labeling of animal foods that contain raw meat or other raw animal tissues (28). The FDA has issued numerous public health advisories related to commercial raw pet food products due to contamination with bacterial pathogens in recent years, including instances where contaminated food was associated with illness in pets (29–33), and raw pet food exposure has also been linked to human illness (25, 34). Raw diets present a human health risk to those who handle raw pet food products directly, are exposed to environmental contamination from improper preparation or handling, or have close contact with pets who consume these products (3, 9). Despite these risks, previous research suggests that the majority of owners who feed raw pet diets perceive the risk of foodborne illness associated with this practice to be low (12, 15).

Given the risks of zoonotic and foodborne illness associated with raw feeding and the importance of appropriate food handling practices, it is essential that those in contact with these products have access to credible information from sources they perceive as reliable (12, 15). Animal and pet feed stores represent settings where food safety information can be provided to both pet owners and store employees. However, while numerous studies have examined food safety perceptions and practices of pet owners (11, 12, 14, 15, 24, 35), information on customer education policies among pet food retailers is scarce, and there is a lack of information on knowledge and sanitation practices of animal and feed store employees. One previous study investigating raw pet food retailers in Minnesota reported a lack of communication regarding risks of foodborne illness and food handling practices at the time of purchase of raw food products, as well as poor hazard labeling of these products (21). Understanding knowledge, practices, and policies in animal and pet feed stores is essential to guide the development of educational outreach strategies for these settings. Therefore, the objectives of this study were to (1) investigate knowledge, sanitation practices, and workplace policies related to handling animal food and treats among employees of pet food retailers in the United States, and (2) determine whether differences exist between employees of stores that sell raw pet food products and those that do not.

## 2 Materials and methods

### 2.1 Ethics approval

This study was approved by the University of Tennessee Institutional Review Board (Number: UTK IRB-23-07792-XM).

### 2.2 Survey development and dissemination

Data for this study were obtained through a survey of pet and animal feed store employees across the United States conducted by the University of Tennessee Center for Agriculture and Food Security and Preparedness (CAFSP), part of the Tennessee Integrated Food Safety Center of Excellence. The survey evaluated current sanitation practices, training, and knowledge of disease risk related to animal husbandry and food handling at the respondent’s workplace. Survey creation and design were performed using QuestionPro Research Edition software (36).

Pet and feed stores across the US were identified by searching for stores that posted their email addresses online. The current study reports the results of a revised version of the original survey instrument, which was disseminated to these stores via email and in-store flyers and had a completion rate of 9.1% (16/176). The final, revised survey was re-distributed using several methods to improve response and completion rates. In addition to email distribution, store managers were contacted by phone to seek participation. After obtaining the store manager’s permission, survey packets were hand-delivered or mailed to pet and feed stores. Existing CAFSP contractors helped to identify pet and feed stores in their area and deliver survey packets to store managers. Student workers at other Centers of Excellence also assisted in identifying stores and distributing the surveys. Surveys were distributed to pet and feed stores in the following states: Alabama, Arizona, California, Colorado, Florida, Georgia, Iowa, Indiana, Illinois, Kansas, Kentucky, Massachusetts, Minnesota, North Carolina, Ohio, Oregon, Pennsylvania, Tennessee, Texas, Washington, and Wisconsin. A total of 451 emails with the QR code and link to the revised survey were sent, and 267 of these surveys were opened and viewed.

Survey packets contained a flyer detailing three ways employees could access the survey: (1) using a QR code, (2) typing an address into their web browser, or (3) completing a paper copy. Postage-paid envelopes were provided for employees who completed surveys by hand. A entry for a voluntary gift card drawing and movie theater candy were provided as incentives for completing the survey. All incentives were purchased using non-grant-related funds. The survey instrument consisted of 33 questions in the following formats: multiple-choice (single and multiple responses), 5-point Likert-type scales (always to never), and 7-point Likert-type scales (strongly agree to strongly disagree). The final survey instrument used to obtain the results presented in this study is provided in the Supplementary material and has been published previously (37). Surveys were completed between July and October 2021.

### 2.3 Statistical analysis

Analysis and visualization of survey data were performed using R version 4.4.0 (38). Differences in the distribution of non-ordered categorical variables between employees of raw food retailers and those of stores that did not sell raw food were assessed using Chi-square tests, or Fisher’s exact tests where indicated based on sample size and expected cell counts. Wilcoxon rank sum tests were used to assess for significant differences between the two groups due to the non-normal distribution of ordinal variables. Adjustment for multiple comparisons was performed using the Benjamini-Hochberg method for controlling the false discovery rate (39). For questions with missing respondents or where respondents could select “Not applicable” (NA) as an answer, Chi-square or Fisher’s exact tests were used to assess whether the proportion of missing and NA values differed between groups. A *p*-value < 0.05 was considered statistically significant.

## 3 Results

Of the 267 respondents who viewed the revised survey, 221 started the survey. A total of 206 surveys (77.2% of surveys that were viewed and 93.2% of those that were started) were completed. Completed surveys were obtained from pet and feed store employees from states in the Southeast (Alabama, Florida, Georgia, Kentucky, North Carolina, Tennessee), Midwest (Illinois, Kansas, Minnesota), Southwest (Arizona, Texas), and Western US (California, Colorado, Oregon, Washington). One hundred and ninety-eight survey respondents reported working in a store that sells pet or animal feed and were included in subsequent analyses. Among these participants, 21.7% (43 respondents) reported working at a store that sells raw dog or cat food. No significant differences in the proportion of “Not Applicable” or missing values were identified between raw food sellers and non-raw food sellers. Eighty-three respondents (41.9%) reported working at a store that was part of a regional or national chain, 50 (25.3%) worked at a store that was part of a local chain (2 stores or more in one town or city), and 58 (29.3%) worked at an independent store (1 location only). The remaining respondents either indicated that they were not sure of their workplace type (1.5%) or did not answer the question (2.0%).

### 3.1 Handwashing, disinfection, and use of personal protective equipment

Most respondents [84.8%; 95% confidence interval (CI): 79.2, 89.2%] reported that their workplace had general handwashing policies, such as requiring employees to wash their hands before and after work, after breaks, and after using the restroom (Table 1). There was no significant difference between stores that sold raw pet food and those that did not [odds ratio (OR) = 1.43; 95% CI: 0.54, 4.54; χ^2^ = 0.2381; *p* = 0.7800]. Similarly, there was no significant difference between the two groups with respect to specific workplace handwashing policies for employees when handling animal food or treats (OR = 2.55; 95% CI: 1.23, 5.24; χ^2^ = 6.9009; *p* = 0.0517).

**Table 1:** Handwashing policies, training, and educational materials reported by employees of raw pet food retailers and non-raw pet food retailers.

Variable	Raw food retailers		Non-raw food retailers		Total	*p*-value
	Percent (95% CI^1^)	Num./Total	Percent (95% CI)	Num./Total	Percent (95% CI)	
Workplace has general handwashing policies	88.4 (75.5, 94.9)	38/43	83.4 (77.3, 88.8)	130/155	84.8 (79.2, 89.2)	0.7800
Workplace has specific handwashing policies for handling animal food or treats	41.9 (28.4, 56.7)	18/43	21.9 (16.1, 29.1)	34/155	26.3 (20.6, 32.8)	0.0517
Respondent received training on handwashing/sanitizing	51.3 (36.2, 66.1)	20/39	47.2 (39.2, 55.4)	67/142	48.1 (40.9, 55.3)	0.7800
Respondent received training on disease risk related to handling animal food	34.9 (22.4, 49.8)	15/43	22.6 (16.7, 29.8)	35/155	25.3 (19.7, 31.7)	0.2008
Workplace displays posters about disease risk from handling animal food	30.2 (18.6, 45.1)	13/43	16.8 (11.7, 23.4)	26/155	19.7 (14.8, 25.8)	0.2008
Workplace provides take-home information to customers about disease risk from handling animal food	23.2 (13.2, 37.7)	10/43	22.6 (16.7, 29.8)	35/155	22.7 (17.4, 29.1)	> 0.999

1 Confidence interval.

Just under half (48.1%, 95% CI: 40.9, 55.3%) of feed store employees who responded to the survey reported that they had received training from their workplace on proper handwashing or sanitation of their hands within the last three years. There was no significant difference between employees of raw food retailers and those that did not sell raw food (OR = 1.18; 95% CI: 0.58, 2.42; χ^2^ = 0.2059; *p* = 0.7800). Similarly, there was no significant difference between the two groups with respect to training related to disease risk associated with handling animal food or pet treats (OR = 1.84; 95% CI: 0.86, 3.80; χ^2^ = 2.6994; *p* = 0.2008), and just 25.3% (95% CI: 19.7, 31.7%) of respondents reported receiving this training at their workplace.

**Figure 1:**
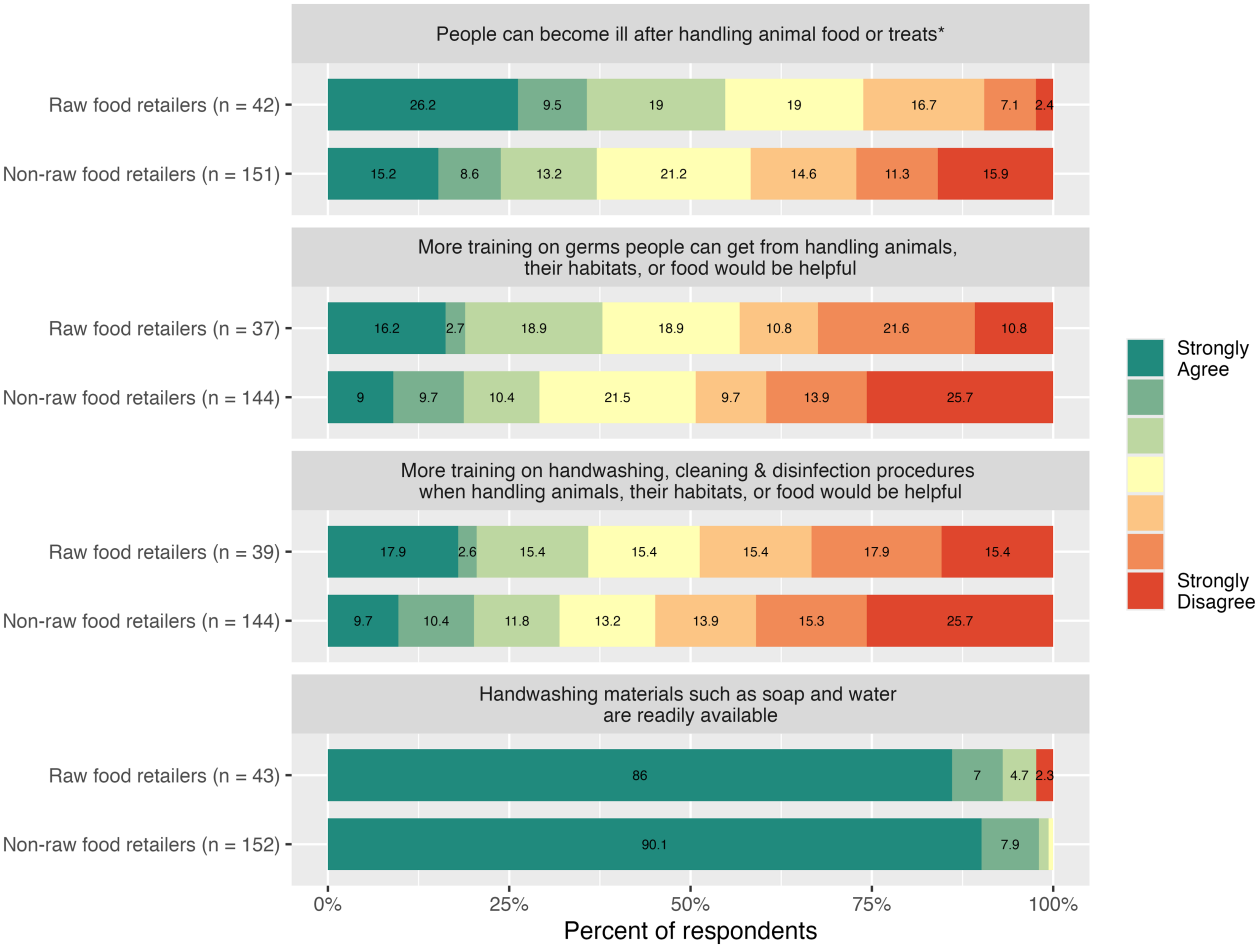
Knowledge and attitudes among employees of raw food retailers and non-raw food retailers. *The asterisk denotes a significant difference between employees of raw food retailers and non-raw food retailers based on the Wilcoxon rank sum test.

**Figure 2:**
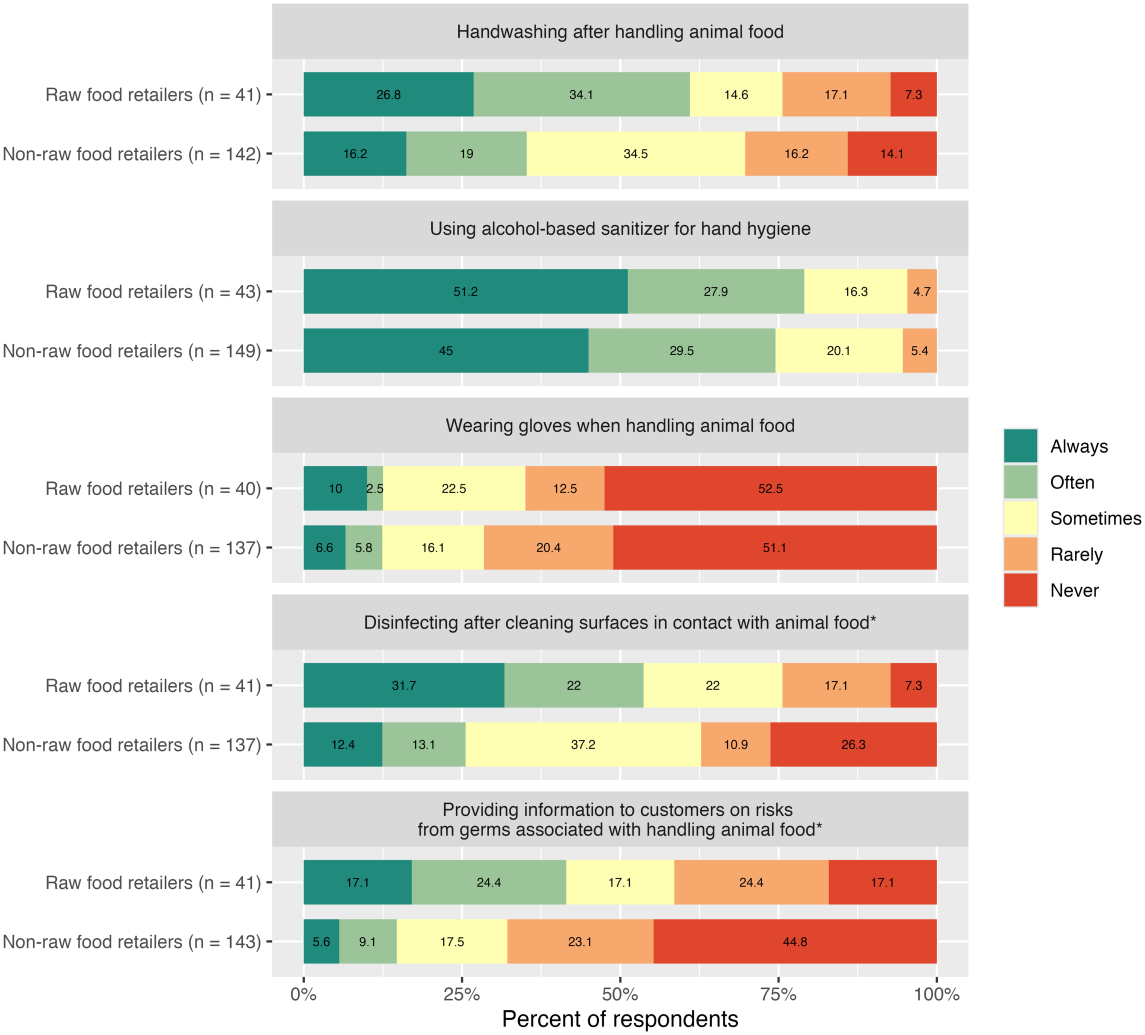
Frequency of sanitation and customer education practices among employees of raw food retailers and non-raw food retailers. *The asterisk denotes a significant difference between employees of raw food retailers and non-raw food retailers based on the Wilcoxon rank sum test.

The availability of handwashing materials did not differ significantly between groups (median difference = 5.37 × 10^−6^; 95% CI: −2.89 × 10^−5^, 4.32 × 10^−5^; *W* = 3415.5; *p* = 0.5061), and the majority of respondents (89.2%) strongly agreed that soap and water were regularly available at their workplaces (Figure 1). There was also not a statistically significant difference in the frequency of handwashing after handling animal food or treats between the two groups (median difference = −1.00; 95% CI: −1.00, −2.56 × 10^−5^; *W* = 2,254; *p* = 0.0542). Notably, 29.0% reported that they rarely or never washed their hands after handling food or treats, while 41.0% always or often washed their hands (Figure 2). Similarly, there was no significant difference between employees of stores that sold raw food and those that did not in the frequency with which they used alcohol-based hand sanitizer (median difference = 5.20 × 10^−6^; 95% CI: −5.54 × 10^−6^, 3.08 × 10^−5^; *W* = 2,977; *p* = 0.5061). Most respondents (75.5%) reported that they often or always used an alcohol-based sanitizer for hand hygiene in their workplace.

Approximately half of feed store employees who responded to the survey (51.4%) reported never wearing gloves when handling animal food or treats, and just 12.4% reported often or always wearing gloves. There was no significant difference between employees of stores that sold raw food and those that did not (median difference = −2.69 × 10^−5^; 95% CI: −3.24 × 10^−5^, 2.58 × 10^−5^; *W* = 2,690; *p* = 0.8508). However, there was a significant difference in the use of disinfectant after cleaning surfaces in contact with animal food between the two groups, with employees of raw food retailers tending to report more frequent disinfectant use (median difference = −1.00; 95% CI: −1.00, −1.89 × 10^−5^; *W* = 1900.5; *p* = 0.0054). About half (53.7%) of raw food sellers reported often or always using disinfectant after cleaning surfaces in contact with animal food, while 24.4% rarely or never disinfected after cleaning. In comparison, 25.5% of those who did not sell raw food reported disinfecting often or always, and 37.2% rarely or never used disinfectant.

### 3.2 Knowledge and attitudes of animal feed store employees

Perceived disease risk associated with handling animal food differed significantly between employees of raw food retailers and those who did not sell raw food (median difference = −1.00; 95% CI: −2.00, −6.23 × 10^−6^; *W* = 2,376; *p* = 0.0360). Just over half (54.8%) of those who sold raw food agreed that people could become ill after touching or handling animal food or pet treats, while 26.2% disagreed (Figure 1). Among employees of stores that did not sell raw food, 37.1% agreed that people could become ill after handling animal food, and 41.7% disagreed. However, the two groups did not significantly differ in attitudes toward additional information or workplace training on disease risk (median difference = −1.29 × 10^−5^; 95% CI: −1.00, 3.50 × 10^−5^; *W* = 2,301; *p* = 0.3521). Overall, 30.9% of animal feed store employees agreed that more information or training on the risk of disease associated with handling live animals, their habitats, or animal food would be helpful. Similarly, the two groups did not significantly differ in attitudes toward additional workplace training on handwashing, cleaning, and disinfection (median difference = −2.42 × 10^−5^; 95% CI: −1.00, 2.10 × 10^−5^; *W* = 2503.5; *p* = 0.4406). About a third of respondents (32.8%) agreed that more information or training on handwashing, cleaning, and disinfection procedures when handling animals, their habitats, or food would be helpful.

### 3.3 Customer education practices and materials

There was a significant difference in customer education practices between employees of raw food retailers and those who did not sell raw food (median difference = −1.00; 95% CI: −1.00, −6.60 × 10^−6^; *W* = 1809.5; *p* = 0.0009). This was assessed in relation to any kind of animal food or treat, with the question “Do you provide information to customers on risks from germs associated with handling of animal food or pet treats?”. Among employees of raw food retailers, 41.5% reported that they often or always provided customers with information on disease risks associated with handling of animal food or pet treats, and 41.5% rarely or never provided this information (Figure 2). In comparison, 14.7% of employees of stores that did not sell raw food often or always provided customers with information on disease risks, while the majority (67.8%) rarely or never provided this information to customers. In terms of specific educational materials, there were no significant differences in the proportion of workplaces that displayed posters or other signage (OR = 2.15; 95% CI: 0.96, 4.65; *χ*^2^ = 3.0508; *p* = 0.2008) or provided take-home information to customers about disease risks associated with handling animal food (OR = 1.05; 95% CI: 0.45, 2.29; *χ*^2^ < 0.0001; *p* > 0.999). The survey questions relating to educational signage and take-home information related to all animal foods, and did not specifically refer to raw food items. Overall, 19.7% (95% CI: 14.8, 25.8%) of respondents reported that their workplace displayed posters or other signage, and 22.7% (95% CI: 17.4, 29.1%) reported that handouts or other take-home information about the risks of illness associated with handling animal food or pet treats were provided to customers (Table 1).

## 4 Discussion

This study investigated knowledge, sanitation practices, and workplace training practices related to handling animal food and treats among employees of pet food retailers in the United States and identified differences between employees of stores that sell raw pet food products and those that do not. Employees of raw food retailers reported disinfecting after cleaning surfaces in contact with animal food more frequently than employees of stores that did not sell raw food, but there were no significant differences in frequency of handwashing or glove use. However, knowledge gaps related to foodborne and zoonotic disease risk, as well as gaps between recommended and reported food safety practices, were identified among both groups.

Notably, just 26.8% of those who sold raw food and 16.2% of those who did not sell raw food reported that they always washed their hands after handling food or treats. This finding is in line with research that investigated handwashing practices among human food service workers, and reported that restaurant employees made handwashing attempts after 32% of activities for which hand washing is recommended (40, 41). While reported levels of non-compliance with hygiene recommendations in the retail food service industry vary widely based on sector and type of activity, research suggests that handwashing is not preformed frequently enough, including during preparation of raw animal products (40, 42). In the pet/animal food store setting, effective hand hygiene is crucial for employees who handle raw food products, as these products have not undergone heat treatment to reduce pathogen loads and may be contaminated with organisms such as *Salmonella*, *L. monocytogenes*, *E. coli*, and others (16–21, 25, 26). However, the U.S. Food and Drug Administration and veterinary professionals also recommend handwashing with soap and water before and after handling heat-treated pet food or treats (43, 44), since conventionally processed pet foods and nutritional supplements can potentially become contaminated with zoonotic pathogens during production and processing (3, 45, 46). Appropriate handling practices can substantially reduce human exposure to *Salmonella* from contaminated dry food products (46).

Most pet and animal feed store employees in this study reported that handwashing supplies were readily available at their workplace. However, respondents tended to report using alcohol-based sanitizers for hand hygiene more frequently than handwashing, which may reflect the convenience of these sanitizers, particularly when sinks are not present in the immediate vicinity. Working in a section of the store that does not have a sink could be a potential barrier to handwashing after handling animal food items. Evidence from the food service industry indicates that odds of appropriate handwashing are significantly higher when multiple hand sinks are present and when there is a hand sink within the employee’s sight (41). While alcohol-based sanitizers can be an effective alternative for reducing the transmission of pathogenic microorganisms when soap and water are not available, they are not as effective for visibly dirty hands or when the quantity of sanitizer or contact time is inadequate (47). The current study also found that most survey respondents either rarely or never wore gloves when handling animal food products. This could be related to the availability of gloves, as in the retail food service industry, where the presence of glove supplies in food preparation areas is significantly associated with glove use (41).

Inadequate hand hygiene and surface disinfection could lead to cross-contamination of surfaces in pet and animal feed stores, as packaging of food items may become damaged during transport or handling, and animal food products may be contaminated with zoonotic pathogens (3). Although employees of raw food retailers tended to disinfect more frequently after cleaning surfaces in contact with animal food, a considerable portion of both groups (24.4% of employees of raw food retailers and 37.2% of employees of non-raw food retailers) rarely or never disinfected surfaces in contact with animal foods. Disinfection after cleaning debris from surfaces in contact with raw food is critical, as *Salmonella* spp. from contaminated raw food has been shown to persist on both stainless steel and plastic surfaces despite cleaning with soap and water (48). In addition, some food products, particularly treats, have been dried but not cooked, and may not be perceived as “raw” pet food products by store employees and pet owners. These include products such as dried pig ears and rawhide chews, which can be contaminated with *Salmonella* and are often stored unpackaged in bulk containers in retail settings (9, 43). Thus, it is important that employees of pet and animal feed stores, including those that do not report selling raw food products, adhere to recommended practices for cleaning and disinfecting surfaces in contact with pet food.

Implementing and increasing awareness of handwashing policies specifically related to animal food handling has the potential to improve hand hygiene practices in the workplace. While most respondents reported that their workplace had general employee handwashing policies, fewer than half (41.9%) of respondents who worked at raw food retailers and just 21.9% of those who did not sell raw food reported that specific policies related to handling animal food or treats were in place. However, this difference was not statistically significant (*p* = 0.0517). This may, to some extent, be attributed to the effect of sample size on the statistical precision of these estimates. Additional research is warranted to investigate the impact of workplace handwashing policies on employee practices in pet and animal feed store settings. It is also worth noting that less than half of survey respondents had received training on methods for washing or sanitizing their hands within the past 3 years. This suggests that adherence to hand hygiene measures could potentially be improved through ongoing outreach and training, with regular scheduling to reduce knowledge gaps. Research in the food service industry has shown that workers provided with food safety training have higher odds of appropriate handwashing (41). However, findings of the current study indicated that about one-third (32.8%) of respondents agreed that more information or training on handwashing, cleaning, and disinfection procedures when handling animals, their habitats, or food would be helpful. This relatively low perceived need among employees, despite the observed gaps in adherence to recommended handwashing practices, could reflect low perceived risk associated with handling animal food products, as well as factors such as time pressure and convenience (41), which may explain the frequent use of alcohol-based hand sanitizers observed in this study. This suggests that training on handwashing and sanitation procedures may be most effective when implemented as part of a comprehensive strategy that also promotes awareness of potential contamination of animal food products, and targets barriers to adequate hand hygiene. Additional research investigating modifiable factors associated with hygiene practices in the pet and animal feed store setting is warranted to identify such barriers.

Compared to employees of stores that did not sell raw food, a higher percentage of employees of raw food retailers were aware that handling animal food or treats can cause illness in humans. However, despite the observed difference, knowledge gaps related to zoonotic and foodborne disease risk exist among both groups. Just 54.7% of respondents who sold raw food and 37% who did not sell raw food agreed that handling animal food or treats can cause illness in humans. Those with a lower perceived risk of foodborne and zoonotic illness may exhibit lower adherence to food safety practices (12), which may explain the lower frequency of surface disinfection among employees that did not sell raw food in this study. Indeed, dog and cat owners with a lower perceived risk of illness associated with pets and their food are significantly less likely to adhere to recommendations for minimizing the risk of zoonotic disease, such as handwashing after interacting with pets and not allowing pets into human food areas (14). The knowledge gaps identified in the current study may reflect a need for more adequate training and education, as only 25.3% of respondents reported receiving training on disease risk related to handling animal food. Implementing workplace training and educational programs related to zoonotic and foodborne disease risk may impact employee perceptions and ultimately, sanitation and food safety practices. The relatively small number of respondents receiving training across both groups suggests that such training programs are generally underutilized in pet food retail settings, regardless of whether raw food products are sold.

Perceived disease risk may also affect how employees communicate with customers regarding risks associated with handling animal food, although it is important to note that this may also be influenced by other factors such as store policy. Employees of raw food retailers, who tended to report higher perceived risk of illness associated with handling animal food, also provided customers with information on these risks more frequently than those who did not sell raw food. However, a substantial percentage of both groups did not provide such information to customers: 41.5% of employees of raw food retailers and 67.8% of those who did not sell raw food either rarely or never provided customers with information on disease risks associated with handling animal food. In addition, among respondents who worked at stores that sold raw food products, just 30.2% reported that their workplace displayed posters, and 23.2% provided customers with handouts or other take-home information about disease risks associated with handling animal food. This is concerning, given previous reports of contamination of RMBDs with bacterial pathogens and the potential human health risks for those who handle these products and/or have close contact with pets who consume them (16–25). It is apparent from previous research that there are gaps in adherence to recommended food safety practices among owners who feed raw pet food products. For example, although handwashing is recommended after feeding, touching, or being licked by pets that consume raw diets (49), between 5 and 40% of pet owners do not wash their hands after feeding raw diets (12, 14), and the majority of owners who feed raw diets allow their pets to lick them and share sleeping areas (14).

Previous research has identified inadequate or inconsistent food safety information on websites of RMBD manufacturers, and confusion among pet owners who feed RMBDs regarding food safety practices (15). Pet owners often rely on sources other than veterinary professionals for information on raw diets, and researchers have called for new strategies to reach pet owners with credible information that they perceive as reliable and non-judgmental (11, 12, 14, 15). Pet food stores are the most common source for obtaining raw food products (35). A survey of US dog and cat owners indicated that 26% of respondents who fed a raw meat diet had learned about these diets from pet store employees (14). Therefore, expanding customer education in these settings could potentially improve pet owners’ awareness of food safety practices and risks of zoonotic and foodborne illness associated with handling RMBDs, which may not be adequately labeled with respect to food safety hazards (21). Effective customer outreach about the safe handling of conventionally prepared pet food products is also important since the majority of pet owners are unaware that dry pet food products may become contaminated and present food safety risks (14). Improved knowledge and awareness of zoonotic disease risk and food safety practices among pet and animal feed store employees may improve their adherence to sanitation practices and empower them to provide customers with reliable food safety information, potentially impacting food safety practices within and beyond the workplace environment.

### 4.1 Strengths and limitations

To our knowledge, this is the first study to examine workplace training, food safety practices and perceptions, and customer education practices among pet food store employees in the United States and investigate differences between those who sell raw food products and those who do not. Completed surveys were obtained from states in every region except for the Northeastern US. Findings of this study provide useful information to guide educational outreach strategies aimed at reducing zoonotic and foodborne illness associated with commercial pet food products. However, this study was not without limitations. The observed lack of significant differences between the two groups of employees may, in part, reflect the limited sample size, particularly the subgroup of respondents who worked at stores that sold raw pet food. Participation in the survey was voluntary, which could have introduced selection bias if respondent characteristics differed from those of non-respondents. Therefore, the results of this study may not be generalizable to all pet food retail employees in the US. Data were self-reported and could, therefore, be impacted by recall and social desirability biases (50). In addition, it was not possible to account for potential clustering of participant responses within stores in the analysis, because data on the number of survey respondents per store were not available. Information the number of stores per state, population density of the surrounding area, and raw food sales for each store was also not available. Finally, the incidence of zoonotic and foodborne illness associated with handling animal food among pet and animal feed store employees is unknown, and cases may be underreported. Future research is warranted to better characterize disease risk in this population to help inform the development of intervention and outreach strategies.

## 5 Conclusion

The findings of this study suggest there is a need for consistent training and educational opportunities regarding food safety practices and the risk of zoonotic and foodborne illness among employees of pet and animal feed stores. Most respondents lacked guidance from specific workplace policies on handwashing after handling animal food or pet treats, and few had received training on the risk of disease associated with handling animal food and treats. A considerable proportion of respondents did not perceive handling of animal food to be associated with risk of illness in humans, and many did not provide customers with food safety information. While employees of raw food retailers tended to have a higher perceived risk of illness and more frequent adherence to surface disinfection measures than employees that did not sell raw food, gaps in knowledge and recommended practices were identified among both groups. Pet and animal feed stores should implement clear and specific hygiene policies and provide regular employee education about disease risk and the importance of hand hygiene, glove use, and surface disinfection. Expanding customer education practices in these settings may also improve awareness of zoonotic and foodborne illness risks and adherence to appropriate food handling practices among pet owners. By addressing these gaps, pet and animal feed stores can improve workplace safety and hygiene standards to minimize disease risks for employees and customers.

## Data Availability

The raw data supporting the conclusions of this article will be made available by the authors, without undue reservation.
